# Consumer Attitudes towards Technological Innovation in a Traditional Food Product: The Case of Wine

**DOI:** 10.3390/foods10061363

**Published:** 2021-06-12

**Authors:** Adrián Rabadán

**Affiliations:** Higher Technical School of Agriculture and Forestry Engineering, University of Castilla-La Mancha, 02071 Albacete, Spain; Adrian.Rabadan@uclm.es

**Keywords:** product innovation, process innovation, neophobia, food technology neophobia, wine neophobia scale

## Abstract

Food innovation is crucial for food companies in order to produce healthier, safer, and more convenient foods. However, there is a segment of consumers reluctant to accept new foods. This attitude is even more important when those novelties are developed in products such as wine that have habitually relied on heritage and traditional production as their main competitive advantage. In this study, consumer attitudes toward innovation in the wine industry were evaluated by simultaneously considering product neophobia and process neophobia. Based upon a sample of 400 personal interviews with Spanish wine consumers, the results showed that these two aspects of neophobia were uncorrelated, meaning they are useful to measure different aspects of general food neophobia. Cluster analysis showed that four different segments of consumers exist, with different attitudes toward technological innovation in the wine industry. The consumer segment that shows the most positive attitudes toward wine innovation (product and process innovation) is that with the highest income and highest level of education. Moreover, greater involvement with the product (wine) results in lower product neophobia. Therefore, future studies should consider product involvement and exposure to cultural diversity as essential factors when evaluating food neophobia.

## 1. Introduction

The increasing interest of food companies in innovation in the search for competitiveness has encouraged the development of innovations even in food sectors in which tradition has long been established as their main competitive advantage [1]. Few food sectors are as influenced by heritage and tradition as the wine production sector, especially in the traditional wine-growing countries [2]. However, changing consumer tastes and the emergence of nontraditional wine markets worldwide have encouraged wine makers to adapt their products in order to succeed [3,4,5,6]. However, innovation in the production of traditional food products must be evaluated carefully [1,7]. In a study conducted in different European countries, Kühne, Vanhonacker, Gellynck, and Verbeke [1] concluded that consumers were open to accepting innovation in traditional food products as long as the traditional character of those foods was preserved. As a result, even if significant consumers segments are interested in new wines [2,5], these innovations should be considered carefully.

Although innovation in the food sector is mainly devoted to the development of novel products [8], innovation within food companies should be understood as a wider process [9]. Drawing on the Oslo Manual [10], two different types of innovation can be identified: technological and nontechnological innovations. The former refers to the development of new products or production processes, while the latter refers to the innovations that companies implement in their marketing strategies or their management [11]. In the wine sector, technological innovations include a range of possibilities, from the addition of different extracts [5] to the use of fungus-resistant grapes [12] or changes in the wine-making process [13]. On the other hand, nontechnological innovations include changes in marketing strategies [14] or the implementation of voluntary certification schemes [15].

Although food innovation is crucial to meet the increasing demand of safer, healthier, and more convenient foods [16,17], the development of novel products faces the rejection of consumers that perceive risks or disadvantages in these innovative foods [18,19]. Several studies have reported that only a small percentage of launched novel food products succeed, while the vast majority continue to fail [7,20,21,22]. This negative perception increases when production is perceived to be more industrial [23] as food innovation is sometimes associated with processed products and unhealthiness [24]. With this in mind, the development of new food products must consider the effects of so-called Food Neophobia (FN), which was defined by Henriques et al. [25] as the personal reluctance to accept and/or enjoy new or unfamiliar foods.

Due to the direct effect of FN on the success of food innovation, numerous tools have been proposed to evaluate this attitude [26]. However, thirty years after its presentation, the most successful tool to evaluate FN is still the Food Neophobia Scale (FNS) developed by Pliner and Hobden [27]. This scale has been used as the basis for the development of other scales to measure additional aspects of FN. This is the case of the so-called Food Technology Neophobia Scale (FTNS), developed to evaluate consumers’ reluctance toward foods produced using new technologies [28]. Various studies have reported that although the FNS and the FTNS are related, the low correlations found on the scores of the two scales (ranges from −0.12 to 0.33) [19] show that they measure different aspects of FN, supporting the idea of general FN as a complex variable that can only be evaluated using a combination of different instruments.

Wine is a complex product, and, for this reason, consumer wine consumer preferences are influenced by a wide range of factors from the emotional and social to intrinsic quality factors [29,30,31]. The importance attributed to each quality factor, from origin to wine barrel aging, depends on consumer involvement with the product [32] but also on individual attitudes that appear as a result of social background [5]. All these specific characteristics of wine encouraged the creation of a specific Wine Neophobia Scale (WNS) [6] using the existing FNS, to specifically evaluate wine neophobia (WN). Few studies have been developed using the WNS, although there are some noteworthy exceptions. This is the case of the study by Nguyen, Johnson, Jeffery, Danner, and Bastian [5], who, after evaluating the level of WN in Australia, Vietnam, and China, found that WN is country-dependent.

After thirty years of studies on FN, some general conclusions have been reached, while other areas are still open to debate [33]. Knaapila et al. [34] reported that up to two- thirds of the variation in individual FN was genetically determined. Supporting the idea of FN as a personal trait, lower FN scores have been associated with a higher individual openness [35] and greater exposure to cultural diversity [36]. Following this association, higher FN scores have also been reported for individuals living in rural areas [36,37]. In this regard, most of the studies coincide in reporting higher levels of FN in individuals with lower levels of education and income [37,38,39,40,41,42]. However, it is still unknown whether the general conclusions reached for general FN might also apply to WN.

The current study aimed to evaluate consumer attitudes toward innovation in the wine industry, including the combined study of WN and food technology neophobia (FTN). The identification and characterization of consumers with the most positive attitudes toward innovation is crucial to develop strategies that ensure wine companies committed to wine innovation achieve higher profits than penalties as a result of their breaking with the association between wine and tradition and heritage.

## 2. Materials and Methods

### 2.1. Database

The data used in this study were collected using survey personal interviews to consumers when they were about to shop in different establishments. The survey was administered in September 2019 in the cities of Madrid, Leganés, and Toledo (Spain) as representative examples of large, medium, and small-sized towns, respectively. A total of 400 individuals who reported to have drunk wine at least once in the previous month were interviewed. A filter question was used for this purpose. The socioeconomic characteristics of the consumers interviewed are shown in Table 1. The maximum sampling error was below 5.0% for a confidence level of 95.5% (k = 2) under the principle of maximum indetermination (p = q = 50%). To confirm that the survey questions were well designed and were easily understandable, the preliminary questionnaire was administered to 30 wine consumers before the fieldwork.

### 2.2. Methodology

In order to evaluate consumer WN, the Wine Neophobia Scale (WNS) was administered [6] (Table 2). The WNS is an 8-item scale developed to evaluate consumer neophobia toward the acquisition of new wines, based on the well-known FNS originally developed by Pliner and Hobden [27] to evaluate general food neophobia. As the questionnaire was administered in Spanish, the translation proposed by Fernández-Ruiz et al. [43] was used, changing the word “*alimento*” (food) to “*vino*” (wine). In the WNS, consumers are asked to evaluate each of the proposed items on a 9-point Likert-type scale ranging from “strongly disagree” (1) to “strongly agree” (9). The individual score on the WNS for each consumer was calculated as the sum of the 8 items after reversing the negative items (items 2, 4, 5, and 8). As a result, consumers obtaining higher values indicate a higher level of wine neophobia and, as a result, a lower tendency to try new wines. On the other hand, consumers with lower scores are identified as wine neophilics, indicating a higher tendency to try new wines.

To evaluate consumer attitudes toward foods produced using novel technologies, the Abbreviated Food Technology Neophobia Scale (AFTNS) was used [44] (Table 2). The AFTNS is an abbreviated 9-item version of the original 13-item Food Technology Scale proposed by Cox and Evans [28]. The translation to Spanish provided by the study of Schnettler et al. [45] was used. Specifically, consumers were asked to evaluate the 9 proposed items using a 6-point Likert-type scale. Individual scores for each consumer were calculated as the sum of the scores for each item after reversing the negative item (item 5). Within the possible range of the scale (9 to 54), higher values indicate a higher level of food technology neophobia (FTN), meaning consumers with a low tendency to accept products that have been produced using novel technologies (NT).

Additionally, consumers provided information about the importance they attached to different wine attributes (such as price or origin) using a 5-point Likert-type scale ranging from very unimportant (1), neither unimportant nor important (3), to very important (5). To identify the attributes included in the survey, previous studies evaluating factors that determine wine purchase decision-making were used [5,46]. Consumers were also required to indicate the frequency of their consumption of wine (four levels). Using a 9-point Likert-type scale from strongly disagree (1) to strongly agree (9), consumers were also asked to indicate their attitudes toward statements linked to wine innovation and their opinions on statements about individual openness. Moreover, to include information on the consumers’ socioeconomic characteristics, they were asked to provide information about gender, age, highest level of education completed, and net household income.

Similarly to Schnettler, Grunert, Miranda-Zapata, Orellana, Sepúlveda, Lobos, Hueche, and Höger [44], a cluster analysis using hierarchical conglomerates was used with linkage by Ward’s method and the squared Euclidian distance as the measure of similarity between objects [47]. The aim of the segmentation was to identify consumer segments according to consumer scores obtained on the WNS and AFTNS. Four different groups with different attitudes were obtained. To describe the differences between the obtained segments, one-way analysis of variance (ANOVA) with Tukey’s HSD post-hoc (significance level 5%) comparison was used to examine responses about wine attributes and the results of the scales, and Pearson’s chi^2^ test was applied to discrete variables. For the statistical analysis of the data, the Statistical Package for Social Sciences IBM SPSS version 23 was used.

## 3. Results and Discussion

The results show that consumers interviewed in this study are more neophobic than the average consumer in Australia [5]. In this regard, the deeper tradition of Spain as a growing wine country could be the explanation for consumers’ less positive attitude toward the acceptance of novel wines [2]. However, this explanation contradicts the widely accepted idea of FN decreasing with the exposure to product diversity [36]. In European countries in which viticulture is traditional, wine consumers are exposed from birth to a wide range of different wines, yet their level of wine neophobia seems to be higher than in other emerging markets such as Australia. Castellini and Samoggia [2] suggested that in European countries in which viticulture is traditional, consumers are influenced by their countries’ wine traditional heritage but, at the same time, excited about trying novel or innovative wines. To our knowledge, only the study by Nguyen, Johnson, Jeffery, Danner, and Bastian [5] has evaluated the level of wine neophobia in several countries simultaneously, concluding that consumers from Vietnam were more neophobic than those from China and Australia.

The correlation found between the score obtained by consumers on the WNS and the AFTNS was not significant, showing a value within the range reported for previous studies between the FNS and the FTNS [19,28,44,48,49,50,51]. This result confirms the proposed idea of the WNS and FTNS measuring different aspects of FN. Moreover, the average score obtained on the WNS for Spanish consumers is similar to the values reported by Fernández-Ruiz, Claret, and Chaya [43] for general FN using the FNS. In this sense, further studies of the correlation between WN and general FN should be conducted to evaluate whether one could be used as a proxy of the other.

The results of the consumer segmentation analysis using the scores obtained on the WNS and the AFTNS are shown in Table 3. Four different consumer segments comprising a similar number of consumers were obtained. Segment 1 is composed of those reported as wine neophilics as they show the lowest values of WN and TFN. A segment of neophobics was also identified (Segment 4). This segment was formed by consumers that showed the highest values of WN and FTN, meaning they had the lowest interest in trying new wines and also the lowest rate of acceptance of any product produced using innovative technologies. Two consumer segments with intermediate characteristics between these two groups were also identified. Segment 2 was formed by consumers with low values of WN but high values for TFN. Opposite attitudes were reported by those consumers aggregated in Segment 3.

Segments composed of consumers that show lower WN scores (Segments 1 and 2) are also those that report a higher frequency of wine consumption (more than 60% of them consume wine at least once a week). This result supports the idea that a greater exposure to the product, in this case, a higher frequency of wine consumption, is associated with a lower product neophobia [36]. Similar results were obtained by Nguyen, Johnson, Jeffery, Danner, and Bastian [5], who also found a correlation between consumption frequency and WN in different countries. More involved consumers (the group of neophilics and neophilics anti-NT) attach greater importance to most of the wine attributes considered, including grape variety, wine barrel aging, or the presence of quality labels. When purchasing food products, a higher involvement has also been associated with the evaluation of a wider range of attributes in products such as fruits [52]. On the other hand, WN has no significant effect on the importance consumers attach to the price when purchasing wine.

Table 4 shows the attitudes of the reported segments toward the statements linked to the wine purchasing process. Wine neophilic segments showed more positive attitudes toward online wine shopping. Regarding wine distribution, Casali et al. [53] found that innovative wine companies opt more for direct distribution channels. Our results suggest that innovative consumers could shop at innovative wineries using online sales as a direct selling channel. Consumers that are more open to wine innovation also place more trust in wine advertising more and pay greater attention to bottle aesthetics and winery image when purchasing wine. This encourages wine makers to develop nontechnological innovations (marketing and organizational innovations) also directed to these segments of wine neophilics.

Table 5 shows the socioeconomic characteristics of each designated segment. Significant differences were found between consumer segments regarding income and education, but not according to gender or age. No effect of gender on consumer neophobia was expected as, although a study developed in very specific populations reported some effects [54], large-scale studies developed in countries such as Portugal [55] or Sweden [56] have concluded that there is no association between gender and FN.

Although significant differences for age were not found, the age profile of the segment of neophilic consumers showed slight differences compared to the profile of neophobics. For example, the number of consumers over 65 years old was twice as large in the neophobic segment as in the neophilic segment. In this sense, Castellini and Samoggia [2] reported that in countries with a long tradition of wine consumption, young consumers show more positive attitudes toward wine innovation [2]. However, in one of the most comprehensive studies about evolution of FN with age, Dovey et al. [57] concluded that FN reaches its highest point in childhood and then decreases during adolescence, is stable during adulthood, and then increases slightly in older ages as health problems appear. As wine consumption is mainly concentrated in adulthood, the reported absence of age-related differences between segments is consistent with the model proposed by Dovey, Staples, Gibson, and Halford [57].

On the other hand, significant differences were found for the variables of education and income. The segment of neophilic consumers was composed of the consumers with the highest income and level of education, while the segment of neophobic consumers showed the lowest income and the lowest education level. Currently, there is a consensus on the negative association between individuals’ FN scores and higher education level and income [33,37,40]. According to our results, this association between lower income and education and higher neophobia also appears when WN is considered instead of general FN. Flight, Leppard, and Cox [36] explained this association by considering that exposure to greater cultural diversity is expected for consumers with a higher socio-economic status, which results in lower neophobia.

In a recent study, Rabadán and Bernabéu [33] suggested that globalization tends to reduce the level of FN worldwide as exposure to different cultures increases [36]. The association between lower FN and higher individual openness was initially proposed by Knaapila, Silventoinen, Broms, Rose, Perola, Kaprio, and Tuorila [35]. To evaluate the association between personal openness and technological innovation in the wine sector, consumers were asked to evaluate their degree of agreement with different statements (Table 6). The results showed that according to the statistical differences reported for some items, a higher openness could be attributed to the less neophobic segments, i.e., they are more willing to travel and discover new cultures. However, in the statements covering the self-reported attachment to tradition, a clear pattern did not appear.

## 4. Conclusions

This study shows that wine consumers show different levels of WN and FTN. This result supports previous findings stating that WN and FTN are useful to measure different aspects of FN. Beyond this, the study suggests that wine consumers with a higher income and level of education tend to be more open to trying new wines. This confirms that the idea of lower education and income leading to higher FN scores is also applicable to the wine sector. Results also state that WN reduces as product involvement increases. For this reason, neophilic consumers consume wine more frequently and consider a wider range of attributes when purchasing wine.

Regarding practical implications, obtained results encourage wine makers to develop new wines and novel production techniques, focusing on consumers with higher socioeconomic status. Within wine attributes, wine makers should pay special attention to the type of wine, the wine barrel aging, and the brand image as these are the key attributes considered by this consumer segment in the purchasing process. Wine makers should also consider that consumer segments eager to try innovative wines exist; however, not all of them are open to the same kinds of innovations, and wines including specific innovations intended for specific consumers segments would need to be developed.

Although wine is considered a traditional food product deeply linked to heritage, wine innovation is crucial to ensure increased demand, mainly in the traditional wine producing countries of Europe, and specifically in Spain, where wine consumption is significantly lower than in surrounding countries. Consumers are accustomed to continuous innovations in the food sector, and the wine sector cannot only rely on their strong connections with *terroir* and culture to survive. Moreover, severe global competition and the positive attitudes toward the innovation of consumers in emerging markets encourage these innovations.

There were some limitations to this work. First, surveys were only conducted in three locations, while the results have been extrapolated to the whole country. It should also be considered that due to the sampling method, some bias could appear. In addition, the use of different Likert-type scales may have led to confusion for some of the interviewees. Second, in market research, there may be a difference between what consumer respondents say and what their real attitudes are. Third, it should be considered that the present study has only evaluated consumer attitudes toward technological innovations (product and process), while nontechnological innovations have been neglected.

Future studies should examine the specific innovations that wine consumers are more willing to accept. Recent knowledge suggests that innovations considered too radical could face rejection even among more pro-innovation consumers. In this regard, specific studies about consumers’ acceptance of nontechnological innovations, including marketing and organizational innovations, should be developed. 

## Figures and Tables

**Table 1:** Socioeconomic characteristics of the sample (%).

Variables	Percentage (%)
Gender	
Men	56.9
Women	43.1
Age (in years)	
18–24	14.0
25–34	21.0
35–49	20.0
50–64	25.0
>65	20.0
Education level	
Elementary	28.5
Secondary	39.5
University	30.6
Postgraduate	1.5
Net monthly family income	
<EUR 1500	17.5
EUR 1500–2100	22.8
EUR 2101–3000	35.6
>EUR 3000	24.2

**Table 2:** Items from the Wine Neophobia Scale (WNS) and the Abbreviated Food Technology Neophobia Scale (AFTNS).

WNS		AFTNS	
Item	Statements	Item	Statements
1	I like going to places serving wines from different countries. (R)	1	New foods are not healthier than traditional foods.
2	I will drink almost any wine. (R)	2	The benefits of new food technologies are often grossly overstated.
3	I am afraid to drink wines I have never had before.	3	There are plenty of tasty foods around, so we do not need to use new food technologies to produce more.
4	At social gatherings, I will try a new wine. (R)	4	New food technologies decrease the natural quality of food.
5	I like wines from different countries. (R)	5	New food technologies are unlikely to have long-term negative health effects. (R)
6	If I do not know what wine it is, I won’t try it.	6	New food technologies may have long-term negative environmental effects.
7	I do not trust new wines.	7	It can be risky to switch to new food technologies too quickly.
8	I am constantly trying new and different wines. (R)	8	Society should not depend heavily on technologies to solve its food problems.
		9	There is no sense trying out high-tech food products because the ones I eat are good enough.

**Table 3:** Consumer segmentation according to Wine Neophobia Scale (WNS) and Abbreviated Food Technology Scale (AFTNS).

	Segment 1 Neophilics (*n* = 102)	Segment 2 Neophilics Anti-NT (*n* = 93)	Segment 3 Neophobics Pro-NT (*n* = 112)	Segment 4 Neophobics (*n* = 93)
Scales				
WNS (*p* = 0.000)	23.59 ^b^	24.06 ^b^	40.34 ^a^	41.33 ^a^
AFTNS (*p* = 0.000)	18.79 ^c^	33.25 ^a^	21.45 ^b^	34.49 ^a^
Consumption frequency (%)				
A few times per week	20.6	21.5	9.8	18.3
Once per week	43.1	40.9	18.8	20.4
Once per two weeks	8.8	15.1	15.2	15.1
Once per month	27.5	22.6	26.3	46.2
Wine attributes				
Type (*p* = 0.007)	4.32 ^ab^	4.59 ^a^	4.15 ^b^	4.48 ^a^
Grape variety (*p* = 0.000)	3.53 ^a^	3.66 ^a^	2.71 ^b^	3.01 ^b^
Barrel aging (*p* = 0.000)	4.09 ^a^	4.01 ^a^	2.95 ^b^	3.31 ^b^
Price (*p* = 0.068)	3.77	4.22	3.95	4.12
Origin (*p* = 0.001)	3.63 ^ab^	3.88 ^a^	3.31 ^c^	3.32 ^bc^
Brand image (*p* = 0.000)	3.94 ^a^	3.90 ^a^	3.27 ^b^	3.56 ^ab^
Bottle aesthetics (*p* = 0.010)	3.16 ^ab^	3.30 ^a^	2.71 ^b^	2.85 ^ab^
Quality labels (*p* = 0.001)	3.69 ^a^	3.67 ^a^	3.04 ^b^	3.25 ^b^
Organic (*p* = 0.000)	2.36 ^a^	2.45 ^a^	1.94 ^b^	1.75 ^b^

The means of the values for the consumers in each segment are shown for the scales and for the wine attributes. Different letters in the same row mean significant differences for scales and wine attributes (*p* < 0.05). Chi-squared values for wine consumption frequency are: X^2^ = 44.101, df = 9, *p* = 0.000.

**Table 4:** Impacts of WN and FTN on wine consumers’ opinions toward statements linked to wine innovation.

	Neophilics (*n* = 102)	Neophilics Anti-NT (*n* = 93)	Neophobics Pro-NT (*n* = 112)	Neophobics (*n* = 92)
I would like to buy more wine online (*p* = 0.000)	3.37 ^a^	2.52 ^b^	1.72 ^c^	1.42 ^c^
I would like to have my favorite wine accessible in more retailers (*p* = 0.088)	1.63	2.13	1.63	1.72
I trust advertising about new wines (*p* = 0.000)	7.08 ^a^	6.75 ^a^	5.13 ^b^	5.54 ^b^
Wine bottle aesthetics are very important to me (*p* = 0.000)	6.02 ^a^	6.02 ^a^	3.96 ^c^	5.04 ^b^
I value the corporate image of the winery when purchasing a wine (*p* = 0.000)	7.03 ^a^	6.71 ^a^	4.59 ^c^	5.52 ^b^

The means of the values for the consumers in each segment. Likert 9-point scale from strongly agree (1) to strongly disagree (9). Different letters in the same row mean significant differences in the consumers’ opinions (*p* < 0.05).

**Table 5:** Socioeconomic characteristics of the segments (%).

	Neophilics	Neophilics	Neophobics	Neophobics
	(*n* = 102)	Anti-NT	Pro-NT	(*n* = 92)
		(*n* = 92)	(*n* = 112)	
Gender (%)				
Men	65.7	57	50	55.9
Women	34.3	43	50	44.1
Age (%)				
18–24	11.8	11.8	17	15.1
25–34	20.6	23.7	20.5	19.4
35–46	25.5	22.6	17	15.1
50–64	28.4	23.7	24.1	23.7
65+	13.7	18.3	21.4	26.9
Education level (%)				
Elementary	20.6	29	28.6	36.6
Secondary	41.2	35.5	48.2	31.2
University	34.3	33.3	23.2	32.3
Postgraduate	3.9	2.2	0	0
Net family income (%)				
<EUR 1500	9.3	33.8	7.2	21.9
EUR 1500–2100	13.5	9.3	38.1	29.3
EUR 2101–3000	39.6	32.6	41.2	28
>EUR 3000	37.5	24.4	13.4	20.7

Chi-squared values for the socioeconomic variables are: gender, X^2^ = 5.42, df = 3, *p* = 0.143; age, X^2^ = 26.79, df = 12, *p* = 0.571; education level, X^2^ = 18.703, df = 9, *p* = 0.028; net family income, X^2^ = 72.580, df =12, *p* = 0.000.

**Table 6:** Impacts of WN and FTN on statements about individual openness.

	Neophilics (*n* = 102)	Neophilics Anti-NT (*n* = 92)	Neophobics Pro-NT (*n* = 112)	Neophobics (*n* = 92)
I consider myself a traditional person (*p* = 0.006)	5.58 ^ab^	6.30 ^a^	4.93 ^b^	5.89 ^ab^
Religion is very important to me (*p* = 0.001)	4.84 ^ab^	3.76 ^b^	5.31 ^a^	4.99 ^ab^
I support the traditional values of the family (*p* = 0.000)	5.01 ^b^	6.48 ^a^	6.85 ^a^	7.04 ^a^
I like traveling and discovering new cultures (*p* = 0.000)	8.15 ^a^	8.04 ^a^	6.58 ^b^	6.61 ^b^
I believe that immigration is positive for my country (*p* = 0.351)	4.71	5.06	4.52	4.59

Means of the values for the consumers in each segment. Likert 9-point scale from strongly agree (1) to strongly disagree (9). Different letters in the same row mean significant differences in the consumers’ opinions (*p* < 0.05).

## Data Availability

Data will be available under request.
